# Primary care contact, clinical management, and suicide risk following discharge from inpatient mental health care: a case–control study

**DOI:** 10.3399/BJGPO.2023.0165

**Published:** 2024-10-30

**Authors:** Rebecca Musgrove, Matthew J Carr, Nav Kapur, Carolyn A Chew-Graham, Faraz Mughal, Darren M Ashcroft, Roger T Webb

**Affiliations:** 1 Division of Psychology and Mental Health, University of Manchester, Manchester, UK; 2 National Institute for Health and Care Research (NIHR) Greater Manchester Patient Safety Research Collaboration, University of Manchester, Manchester, UK; 3 Manchester Academic Health Science Centre, Manchester, UK; 4 Centre for Pharmacoepidemiology and Drug Safety, University of Manchester, Manchester, UK; 5 Mersey Care NHS Foundation Trust, Prescot, UK; 6 School of Medicine, Keele University, Newcastle-under-Lyme, Staffordshire, UK; 7 Midlands Partnership University NHS Foundation Trust, Stafford, UK; 8 NIHR Applied Research Collaboration West Midlands, UK

**Keywords:** primary health care, suicide, psychiatric discharge, mental health

## Abstract

**Background:**

Evidence is sparse regarding service usage and the clinical management of people recently discharged from inpatient psychiatric care who die by suicide.

**Aim:**

To improve understanding of how people discharged from inpatient mental health care are supported by primary care during this high-risk transition.

**Design & setting:**

A nested case–control study, utilising interlinked primary and secondary care records in England for people who died within a year of discharge between 2001 and 2019, matched on age, sex, practice-level deprivation, and region with up to 20 living discharged people.

**Method:**

We described patterns of consultation, prescription of psychotropic medication, and continuity of care for people who died by suicide and those who survived. Mutually adjusted relative risk estimates were generated for a range of primary care and clinical variables.

**Results:**

More than 40% of patients who died within 2 weeks of discharge and >80% of patients who died within 1 year of discharge had at least one primary care consultation within the respective time periods. Evidence of discharge communication from hospital was infrequent. Within-practice continuity of care was relatively high. Those who died by suicide were less likely to consult within 2 weeks of discharge (adjusted odds ratio [AOR] 0.61 [95% confidence interval {CI} = 0.42 to 0.89]), more likely to consult in the week before death (AOR 1.71 [95% CI = 1.36 to 2.15]), be prescribed multiple types of psychotropic medication (AOR 1.73 [95% CI = 1.28 to 2.33]), experience readmission, and have a diagnosis outside of the ‘severe mental illness’ definition.

**Conclusion:**

Primary care clinicians have opportunities to intervene and should prioritise patients experiencing transition from inpatient care. Clear communication and liaison between services is essential to provide timely support.

## How this fits in

General practice has a key role to play in preventing suicides among people recently discharged from inpatient psychiatric care. Evidence for post-discharge primary care service utilisation patterns and clinical management is, however, sparse. This investigation of interlinked electronic health records in England revealed that most patients who died by suicide within a year of discharge engaged with primary care services, and that >40% of those who died by suicide within 2 weeks of their discharge consulted with a GP. Evidently, there are opportunities to monitor these patients and intervene during this risky transition period.

## Introduction

Prevention of suicide after discharge from inpatient psychiatric care is a healthcare priority globally.^1–5^ Mental health facilities and the services that they discharge people to, including primary care, have a responsibility to keep patients safe with proactive support and follow-up.^1^ Existing relationships between patients and their GPs can enable continuity of care that may help to reduce post-discharge suicide risk.^6^ GPs have a central role in supporting their patients’ mental health after hospital discharge.^7^ The transfer of information between services at this transition point has also been identified as an important component of continuity.^8^ In England, the National Institute for Health and Care Excellence (NICE) guidance on transition between inpatient mental health settings and the community^1^ includes two primary care specific recommendations: the discharging hospital should 1) consider organising a GP follow-up appointment within 2 weeks of discharge; and 2) ensure that a discharge letter is emailed to the patient’s GP within 24 hours, and a summary sent within a week, subject to the patient’s agreement.

Despite these clinical recommendations, our database search and narrative review of health service support after discharge for people who died by suicide revealed only two studies, from Austria, which reported results specifically on primary care contact for patients who died by suicide post-discharge.^9,10^ Patients who died by suicide within 12 weeks of discharge were more likely to be ‘in current GP treatment’ than those in the general population who died by suicide,^9^ but less likely than their surviving discharged peers to have an appointment made with their GP by the discharging hospital.^10^ Although detailed information about these consultations was unavailable, these studies show that patients discharged from inpatient psychiatric units tend to be in contact with primary care, and suggest the potential protective benefit of the discharging hospital arranging GP appointments post-discharge.

To build on the limited existing evidence base, we assessed variation in primary care utilisation and clinical management between discharged patients who died by suicide within a year of discharge and those who survived. This study aimed to improve our understanding of how people discharged from inpatient mental health care access primary care and to highlight the opportunities that GPs have for providing support during this risky transition period. Specifically, we examined the following: a) frequency and timing of consultations; b) psychotropic medication prescribing; and c) continuity of care (information transfer from hospital to primary care and consulting with the same GP on multiple occasions).

## Method

### Data source and ethical approval

This study utilised the Clinical Practice Research Datalink (CPRD) GOLD and Aurum datasets, comprising general practice data from two electronic patient management systems. Broadly representative of the UK population, the CPRD covers approximately 20% of all people registered with an NHS general practice. CPRD data were linked with the Hospital Episode Statistics Admitted Patient Care (HES APC) dataset, Office for National Statistics (ONS) mortality records, and the 2015 English Index of Multiple Deprivation (IMD) quintiles. The CPRD has ethical approval to support research using anonymised data. The authors do not hold any patient identifiers. These are removed by the statutory body that can receive patient identifiable data, NHS England, before data transfer to protect patient confidentiality. Patient consent was not required as data are collected routinely and anonymised. Any patient may opt out of their data being used in research. See Supplementary Information S1 for further information.

### Study population and design

Adults who died by suicide between 1 January 2001 and 31 May 2019 within a year of discharge from an inpatient psychiatric ward in England comprised the cases in this nested case–control study. Individuals were selected from a cohort of 100 761 discharged patients identified in HES APC records. We have reported the study cohort’s selection procedures previously.^11^ Up to 20 cohort members were selected as controls for each person who died by suicide using incidence density sampling.^12^ Controls were discharged at similar times to their matched cases (±2 years), and matched on sex, year of birth (±2 years), region, and practice-level IMD quintile. The look-back period for measuring clinical variables was from suicide date to discharge date, and the equivalent time elapsed for controls (see Supplementary Information S2 and Supplementary Figure S1). Controls were only selected for people who died after 2-weeks post-discharge to give the opportunity for at least one GP consultation, in line with NICE guidance.^1^

### Classification of outcomes and covariates

Suicides including unnatural deaths of undetermined intent, as is convention among UK researchers,^13^ were identified from ONS mortality records using *International Classification of Diseases, Tenth Revision* (ICD-10) codes X60–X84, Y10–Y34 (Y33.9 excluded), Y87.0, and Y87.2.^14^ Consultation types were limited to face to face, telephone, or remote. Total consultations were limited to one per staff member per day and were grouped by GP and all other staff types (see Supplementary Information S2). To account for different look-back periods among patients, a consultation frequency variable was developed using cumulative consultation numbers on each day after discharge for each discharged cohort member. A variable denoting whether a patient was selected from the Aurum or GOLD datasets was used in the multivariable model to adjust for coding practice differences.

Relational continuity of care, the ongoing relationship between a patient and a practitioner,^8^ was measured during the first 3-months post-discharge. The Usual Provider of Care (UPC) index^15^ was used (see Supplementary Information S2). For consistency, continuity was only estimated for patients with at least a 3-month look-back period. Informational continuity, the sharing of knowledge between the discharging hospital and the patient’s general practice,^8^ was measured via the identification of codes indicating a discharge, and receipt and timing of a discharge summary. Relevant Read and SNOMED CT codes^16^ (primary care coding schemes), and consultation type codes documenting a discharge, were reviewed by CAC-G and FM. Codes entered within 3-months post-discharge were assumed to relate to the index discharge.

Prescriptions for psychotropic medications were extracted using relevant codes. The derived variable counted how many of 10 medication types were prescribed to a patient at any time during the look-back period. Binary variables, pertaining to any prescriptions of tricyclic antidepressants (TCAs), strong opioids, or gabapentinoids, were also generated (see Supplementary Information S2). For patients who died by suicide, underlying and secondary causes of death from ONS data coded as ICD T43.0 (poisoning by TCAs and tetracyclic antidepressants [TeCAs]) were extracted. Inpatient psychiatric readmission was measured in HES APC as an admission under a consultant psychiatrist. Length of stay was pre-calculated in HES data. For code lists, see https://clinicalcodes.rss.mhs.man.ac.uk.

### Data analysis

Analyses were performed using Stata (version 16) software. Patients who died by suicide (cases) were split into the following two groups: those who died within 2 weeks; and those who died during the remainder of the follow-up year. Descriptive demographic and clinical management information was provided for both groups. Unadjusted conditional logistic regression models were fitted for individuals who died after 2-weeks post-discharge to estimate the odds of different clinical management variables for discharged patients who died by suicide compared with living matched controls. A multivariable model, mutually adjusting for these variables, was then developed. Variables included in the multivariable analysis were chosen a priori based on NICE guidance^1^ and previous studies of suicide risk and primary care utilisation.^17,18^ As this analysis was purely descriptive, we did not aim to elucidate causal relationships, and therefore there was no primary exposure. Relational continuity of care was not included in multivariable analysis, as it was only calculated for patients with at least 90 days of look-back.

## Results

### Descriptive information

The records of 613 people who died by suicide within a year of discharge were examined, with 93 (15.2%) deaths occurring within 2-weeks post-discharge (see Supplementary Figure S2). Almost three-quarters (72.4%) of patients who died in this early period were male (Table 1) with a median age of 49 years (IQR 40–58 years) (data not shown). They were mostly discharged after a short inpatient stay. The most frequent diagnoses were anxiety, adjustment, and related disorders, and depression (Table 1). Over two-fifths (41.9%) had at least one consultation in primary care, most of whom (*n* = 28/39) were prescribed psychotropic medication.

**Table 1.** Demographic and clinical characteristics of people who died by suicide in the first 2 weeks after discharge (*N* = 93)

Characteristic		*n*	%
**Sex**			
Male		69	74.2
Female		24	25.8
**Age at discharge, years**			
18–34		9	9.7
35–64		67	72.0
≥65		17	18.3
**Primary diagnosis at discharge^a^**			
Schizophrenia		6	6.5
Bipolar disorder		—	—
Depression		23	24.7
Anxiety, adjustment, and related disorders		30	32.3
Substance misuse		8	8.6
Personality disorders		—	—
All other codes		26	28.0
**≥1 comorbidities at baseline**		21	22.6
**Length of stay, days**			
0–7		41	44.1
8–29		30	32.3
≥30		22	23.7
**Consultation in primary care post-discharge**		39	41.9
**Psychotropic medication types prescribed**			
0		65	69.9
1		9	9.7
2		11	11.8
≥3		8	8.6

^a^Figures for bipolar disorder and personality disorders total fewer than five people each and have therefore been included in ‘all other codes’

Discharged patients who died after the first 2 weeks were on average slightly younger, with a median age of 46 years (IQR 35–58 years) (data not shown), and 31.2% had received a depression diagnosis (Table 2). Over four-fifths (81.5%) had at least one consultation, 63.2% (*n* = 268/424) of whom had their first consultation within 2 weeks. Of those who died by suicide, 36.3% had a high frequency of consultations compared with 29.7% of living control patients; 36.2% were prescribed ≥3 psychotropic medication types compared with 21.5% of living control patients; and 9.2% were prescribed a TCA (Table 1), 25.5% of whom had poisoning by TCAs or TeCAs recorded as a cause of death (data not shown). In the week before death, 31.5% consulted (Table 1). Almost one-third (31.7%) of people who died within a year were readmitted to inpatient psychiatric care.

**Table 2.** Demographic and clinical characteristics of people who died by suicide after 2 weeks but within 1 year of discharge and their corresponding controls who did not die during the equivalent period

		Cases, *n* = 520		Controls, *n* = 8354	
**Characteristic**		***n***	**%**	***n***	**%**
**Sex**					
Male		348	66.9	5601	67.0
Female		172	33.1	2753	33.0
**Age at discharge, years**					
18–34		126	24.2	2080	24.9
35–64		313	60.2	5165	61.8
≥65		81	15.6	1109	13.3
**Primary diagnosis at discharge**					
Schizophrenia		51	9.8	1422	17.0
Bipolar disorder		34	6.5	545	6.5
Depression		162	31.2	1689	20.2
Anxiety, adjustment, and related disorders		87	16.7	822	9.8
Substance misuse		52	10.0	1472	17.6
Personality disorder		26	5.0	223	2.7
All other codes		108	20.8	2181	26.1
**≥1 comorbidities at baseline**		99	19.0	1759	21.1
**Length of stay, days**					
0–7		148	28.5	2304	27.6
8–29		222	42.7	3327	39.8
30–89		126	24.2	2016	24.1
≥90		24	4.6	705	8.4
**Number of psychotropic medication types prescribed**					
0		114	21.9	2376	28.4
1		95	18.3	2234	26.7
2		123	23.7	1950	23.3
≥3		188	36.2	1794	21.5
**Tricyclic antidepressants prescribed**		48	9.2	499	6.0
**Opioids prescribed**		32	6.2	420	5.0
**Gabapentinoids prescribed**		26	5.0	240	2.9
**Timing of first consultation**					
Within 2 weeks		268	51.5	4332	51.9
After 2 weeks		156	30.0	2365	28.3
No consultation		96	18.5	1657	19.8
**Frequency of consultations**					
Low		172	33.1	3275	39.2
Medium		159	30.6	2595	31.1
High		189	36.3	2484	29.7
**Consultation in the week before death by suicide**		164	31.5	1702	20.4
**Readmission to inpatient psychiatric care**		165	31.7	1147	13.7
		**Median**	**IQR**	**Median**	**IQR**
Consultations (all staff types)		4	1-8	3	1–7
Consultations with a GP		2	0–6	2	0–5
Face-to-face consultation		3	1-7	3	1–6
Telephone or online consultation		0	0–1	0	0–1

### Continuity of care

Coded evidence of discharge was identified in 17.2% of patients who died by suicide within 2-weeks post-discharge (Table 3). For those who died 2 weeks or later during the follow-up year, the equivalent was 42.5%. However, a code corresponding to receipt of discharge summary within 7 days was identified in 23.7% of records.

**Table 3.** Informational continuity for those who died by suicide

	Died in first 2 weeks		Died during rest of year	
**Informational continuity**	***n***	**%**	***n***	**%**
Some evidence of discharge in record	16	17.2	221	42.5
Specific discharge summary received	10	10.8	157	30.2
Proportion received in the first 7 days			123	23.7

Of the 322 people who died by suicide after 3 months, 61.2% (versus 49.7% of living control patients) had the minimum two consultations needed to calculate relational continuity. The mean UPC scores were 0.72 (standard deviation [SD] 0.24) for those who died and similar for the living control patients at 0.74 (SD 0.24). Thus, almost three-quarters of GP consultations with discharged patients, whether they died from suicide after 3 months post-discharge or they lived throughout follow-up, were with the patient's most-seen GP (data not shown).

### Primary care contact and clinical management

In unadjusted models, discharged patients who died by suicide were more likely to consult at higher frequency than living control patients (odds ratio [OR] 1.44 [95% confidence interval {CI} = 1.15 to 1.79]), although no discernible difference remained after mutual adjustment for other included variables (Table 4). In the adjusted model, patients who died by suicide were less likely to have had a consultation within 2 weeks of discharge compared with no consultation (adjusted odds ratio [AOR] 0.61 [95% CI = 0.42 to 0.89]). Those who died by suicide were more likely to have a consultation in the week before their death (AOR 1.71 [95% CI = 1.36 to 2.15]) and more likely to be prescribed ≥3 psychotropic medication types (AOR 1.73 [95% CI = 1.28 to 2.33]).

**Table 4.** Unadjusted and adjusted models of primary care use after discharge for those who died by suicide (*n* = 520) compared with their discharged counterparts (*n* = 8354)

		Unadjusted			Fully adjusted^a^		
**Characteristic**		**OR**	**95% CI**	***P*** **value**	**AOR**	**95% CI**	***P*** **value**
**Consultation frequency**							
Low		1			1		
Medium		1.14	0.91 to 1.43	0.25	1.15	0.85 to 1.57	0.36
High		1.44	1.15 to 1.79	**0.001**	1.17	0.84 to 1.64	0.36
**Timing of first consultation**							
Within 2 weeks		1.04	0.81 to 1.34	0.78	0.61	0.42 to 0.89	**0.01**
After 2 weeks		1.09	0.82 to 1.44	0.55	0.71	0.50 to 1.01	0.06
No consultation		1			1		
**Consultation in the week before death by suicide**		1.79	1.47 to 2.18	**<0.001**	1.71	1.36 to 2.15	**<0.001**
**Number of psychotropic medication types prescribed**							
0		1			1		
1		0.89	0.67 to 1.18	0.42	0.91	0.67 to 1.24	0.55
2		1.32	1.01 to 1.73	**0.04**	1.20	0.89 to 1.61	0.24
≥3		2.31	1.78 to 2.98	**<0.001**	1.73	1.28 to 2.33	**<0.001**
**Tricyclic antidepressants prescribed**		1.63	1.19 to 2.24	**0.002**	1.26	0.90 to 1.75	0.18
**Readmission to inpatient psychiatric care**		3.14	2.56 to 3.84	**<0.001**	2.93	2.38 to 3.62	**<0.001**
**Primary diagnosis at discharge**							
Schizophrenia		1			1		
Bipolar disorder		1.70	1.08 to 2.67	**0.02**	1.48	0.94 to 2.35	0.09
Depression		2.64	1.90 to 3.67	**<0.001**	2.28	1.62 to 3.21	**<0.001**
Anxiety, adjustment, and related disorders		2.86	1.99 to 4.10	**<0.001**	2.55	1.75 to 3.72	**<0.001**
Substance misuse		1.02	0.68 to 1.51	0.93	1.01	0.67 to 1.52	0.97
Personality disorders		3.03	1.83 to 5.02	**<0.001**	2.33	1.37 to 3.95	**0.002**
All other codes		1.25	0.88 to 1.78	0.21	1.14	0.79 to 1.63	0.49
**Length of stay, days**							
0–7		1			1		
8–29		1.02	0.82 to 1.27	0.83	1.02	0.81 to 1.28	0.86
30–89		0.92	0.71 to 1.19	0.50	0.88	0.67 to 1.16	0.36
≥90		0.51	0.32 to 0.79	**0.003**	0.58	0.37 to 0.93	**0.02**
**≥1 comorbidities at baseline**		0.77	0.60 to 0.99	**0.04**	0.74	0.58 to 0.96	**0.02**

^a^Also adjusted for source dataset. Bold values indicate statistical significance. AOR = adjusted odds ratio. OR = odds ratio.

### Risk factors related to inpatient care

Compared with patients who were given a primary diagnosis of schizophrenia during their inpatient stay, those with anxiety, adjustment and related disorders, personality disorders, and depression had higher odds of dying by suicide (Table 4). Individuals with a length of stay ≥90 days had just under half the odds of dying by suicide than those who stayed for a week or less (AOR 0.58 [95% CI = 0.37 to 0.93]). There were no identified risk differences between short and medium lengths of stay. Finally, the strongest predictor of suicide was ≥1 readmissions to inpatient psychiatric care before death (AOR 2.93 [95% CI = 2.38 to 3.62]).

## Discussion

### Summary

This study has revealed that most people who died by suicide within a year of discharge from inpatient psychiatric care consulted primary care services during the interim. Even among people who died within 2-weeks post-discharge, >40% had a consultation within that time period. These early deaths were characterised by male sex, middle-age, shorter stays, and diagnoses of anxiety, adjustment, or related disorders, depression, or no formal diagnosis. Evidence of hospital discharge summaries in patients’ general practice notes was sporadic. On average, where continuity could be calculated, most consultations were with the same GP. After adjustment, patients who died between 2 weeks and 1 year were less likely to consult within 2-weeks post-discharge compared with not consulting at all, have more prescribed psychotropic medication types, consult in the week before their death, have an inpatient stay <90 days, and experience inpatient readmission. Finally, those who died by suicide were more likely to have diagnoses of anxiety, adjustment or related disorders, depression, or personality disorders, than schizophrenia.

### Strengths and limitations

Interlinked electronic health records in the CPRD enabled examination of patient trajectories across the transition between secondary and primary care. The dataset size provided sufficient power to examine an outcome that is rare in absolute terms. The study, however, had several limitations. First, all patients had to be registered at a CPRD practice from discharge until suicide date to enable assessment of primary care utilisation. This may have excluded some deaths by suicide for people who changed practice. Further research is needed to understand the trajectories of these patients. The lack of linked up-to-date mental health records meant that it was not possible to identify all post-discharge care received. Finally, data incompleteness in discharge documentation led to the exclusion of informational continuity from multivariable analysis owing to likely undercounting; documents may be recorded in notes or scanned without inputting relevant codes.

### Comparison with existing literature

The proportion of suicides in the first 2-weeks post-discharge is broadly comparable with data collected via the National Confidential Inquiry into Suicide and Safety in Mental Health^19^ and were demographically similar to a study of suicide within 2 weeks of discharge in England.^20^ The finding that people who die by suicide are in contact with primary care after discharge corresponds with the previously described study in Austria^9^ and may partly reflect higher levels of primary care attendance for people with severe mental illness (SMI).^21^ In addition, increased consultation levels have been identified in the week before suicide in the general population,^18,22^ possibly reflecting an increase in help-seeking before suicide. Multiple types of prescribed medication have been found to be associated with an elevated suicide risk.^17^ A range of medications can be important in managing psychiatric conditions, but may indicate higher illness severity.^23^ Suicide risk is likely to be owing to this severity rather than a causal effect of the medication. However, the prescribing of TCAs to almost one in ten of those who died is concerning, particularly as 25% of deaths among those prescribed TCAs were caused, at least partially, by this type of medication. Prescribing of TCAs is cautioned in NICE self-harm guidance owing to their toxicity in overdose,^24^ and GPs need to carefully consider its prescription in this patient group. The significant association observed between readmission and suicide has not been consistently identified.^25,26^ People who died by suicide within 2-weeks post-discharge tended to have a short length of stay in line with other UK findings regarding the early post-discharge period.^20,27^ This may indicate premature discharge in some instances.

### Implications for research and practice

This study identified that there are opportunities for intervention in primary care, especially in the initial post-discharge period. Many recently discharged people are known by their GP,^28^ and relational continuity in this study was found to be relatively high. This may be important in understanding when someone is in suicidal crisis. The difficulty in measuring continuity over a short period limited what can be inferred from our results. Qualitative studies investigating patients’ experiences of continuity in primary care after psychiatric discharge will be important to gain deeper insights. Although data quality precluded detailed analysis, evidence of discharge summaries in patients’ primary care records was infrequent. Further research incorporating reviews of patients’ notes would yield a better understanding of information transfer at this time.

However, there are barriers, including workload, time-limited consultations,^29^ and perceived mental health expertise and confidence,^30^ which may hinder early contact between discharged patients and primary care clinicians.^28,31^ Furthermore, an inpatient stay may disrupt the doctor–patient relationship. Support for GPs and clear, timely communication between services — in line with NICE guidance^1^ — is thus essential. We found an early visit to be associated with a lower suicide risk compared with no visit after adjustment for measures, such as attendance frequency, echoing findings from the previously conducted Austrian study.^10^ We recommend that the discharging hospital arranges a post-discharge appointment in primary care as soon as is possible post-discharge.

Policy in England has targeted people with SMI diagnoses. However, this study has shown that post-discharge, patients with other diagnoses, such as adjustment, anxiety and depression, and personality disorders, have elevated suicide risk. NHS England has recently expanded the SMI definition to include a wider range of conditions.^32^ It should consider applying this definition in the Quality and Outcomes Framework (QOF), a primary care scheme that remunerates a standard of care,^33^ and including a specific indicator relating to transition. It is crucial that primary care teams pay particular attention to these patients. Most recently discharged people do access primary care, and each contact presents an opportunity to reduce suicide risk.

## Data

The clinical codes that were applied are available online at https://clinicalcodes.rss.mhs.man.ac.uk. Data can only be accessed via application to the CPRD.
